# Arabidopsis leucine-rich repeat extensin (LRX) proteins modify cell wall composition and influence plant growth

**DOI:** 10.1186/s12870-015-0548-8

**Published:** 2015-06-24

**Authors:** Christian Draeger, Tohnyui Ndinyanka Fabrice, Emilie Gineau, Grégory Mouille, Benjamin M. Kuhn, Isabel Moller, Marie-Therese Abdou, Beat Frey, Markus Pauly, Antony Bacic, Christoph Ringli

**Affiliations:** Institute of Plant Biology, University of Zürich, Zollikerstr. 107, Zürich, 8008 Switzerland; Department of Plant and Microbial Biology, UC Berkeley, Berkeley, CA 94720 USA; INRA, Institut Jean-Pierre Bourgin, UMR1318 Saclay Plant Sciences, Versailles, 78026 France; AgroParisTech, Institut Jean-Pierre Bourgin, UMR1318 Saclay Plant Sciences, Versailles, 78026 France; ARC Centre of Excellence in Plant Cell Walls, School of Botany, the University of Melbourne, Parkville, Victoria 3010 Australia; Swiss Federal Research Institute WSL, Birmensdorf, 8903 Switzerland; Current address: The New Zealand Institute for Plant & Food Research Limited, Auckland, 1142 New Zealand; Current address: Thermo Fisher Scientific, Neuhofstrasse 11, 4153, Reinach, Switzerland

**Keywords:** Arabidopsis, Cell wall development, Cell wall structures, LRR-extensin, LRX

## Abstract

**Background:**

Leucine-rich repeat extensins (LRXs) are extracellular proteins consisting of an N-terminal leucine-rich repeat (LRR) domain and a C-terminal extensin domain containing the typical features of this class of structural hydroxyproline-rich glycoproteins (HRGPs). The LRR domain is likely to bind an interaction partner, whereas the extensin domain has an anchoring function to insolubilize the protein in the cell wall. Based on the analysis of the root hair-expressed *LRX1* and *LRX2* of *Arabidopsis thaliana*, LRX proteins are important for cell wall development. The importance of LRX proteins in non-root hair cells and on the structural changes induced by mutations in *LRX* genes remains elusive.

**Results:**

The *LRX* gene family of *Arabidopsis* consists of eleven members, of which *LRX3*, *LRX4*, and *LRX5* are expressed in aerial organs, such as leaves and stem. The importance of these *LRX* genes for plant development and particularly cell wall formation was investigated. Synergistic effects of mutations with gradually more severe growth retardation phenotypes in double and triple mutants suggest a similar function of the three genes. Analysis of cell wall composition revealed a number of changes to cell wall polysaccharides in the mutants.

**Conclusions:**

LRX3, LRX4, and LRX5, and most likely LRX proteins in general, are important for cell wall development. Due to the complexity of changes in cell wall structures in the *lrx* mutants, the exact function of LRX proteins remains to be determined. The increasingly strong growth-defect phenotypes in double and triple mutants suggests that the LRX proteins have similar functions and that they are important for proper plant development.

**Electronic supplementary material:**

The online version of this article (doi:10.1186/s12870-015-0548-8) contains supplementary material, which is available to authorized users.

## Background

Characteristic features of plants are cell walls that not only protect each individual cell but also strongly influence plant development. While cell growth is driven by turgor pressure, expansion of the primary cell wall, which resists turgor pressure, is the rate limiting step. The primary cell wall is composed of complex interlinked networks, mainly composed of cellulose, non-cellulosic and pectic polysaccharides such as xyloglucan and heteroxylans, and also structural cell wall proteins. Cell wall expansion therefore requires a constant rearrangement of these supramolecular structures by integrating new material, modifying linkages, and adjusting the composition of the cell wall [1]. Interfering with either the biosynthesis or post deposition modification of cell wall components causes changes in cell wall structure/organisation, which can influence cell growth processes, as exemplified by a number of cell wall mutants of *Arabidopsis thaliana* that show changes in cell morphology (for review, see [2]).

Plants have developed a sophisticated system to monitor cell wall formation in order to respond to changes in cell wall composition [2–5]. Genetic approaches have led to the identification of a number of receptor-like transmembrane proteins that perceive signals from the cell wall and transduce them to the cytoplasm. Wall-associated kinases have a cytoplasmic kinase domain and an extracellular domain that can bind pectin, and serve functions in pathogen response as well as regulation of osmotic pressure [6–9]. *THESEUS1* encodes a CrRLK-like receptor kinase that monitors changes in the cell wall caused by a reduced cellulose content and induces secondary changes in the cell wall such as lignin deposition [10, 11].

Leucine-rich repeat (LRR) proteins have been identified in a number of systems to act as interaction partners in either a signaling cascade or as modulators of protein activity. Polygalacturonase inhibitors (PGIPs) specifically bind polygalacturonases, thereby inhibit their enzymatic function, and thus influence the turnover of pectic polysaccharides [12]. Pathogen-recognizing disease resistance proteins often contain an LRR domain which is thought to interact with a pathogen-induced molecule [13]. On the other hand, the brassinosteroid and auxin binding proteins BRI and TIR1 harbour LRR domains [14, 15], revealing the broad chemical spectrum of potential binding partners of LRR domains. Out of over 200 LRR-receptor proteins encoded in Arabidopsis, some have been shown to be important for cell wall developmental processes. *FEI1* and *FEI2* influence cell wall function and cell growth properties by affecting cell wall composition [16].

LRR-extensin (LRX) proteins are extracellular proteins found in different plant species [17, 18]. LRX proteins contain an N-terminal LRR domain with 10 complete LRRs, and a C-terminal extensin domain with (Ser-Hyp_4_)-containing repetitive motifs typical for this class of HRGPs [19, 20]. While the LRR domain is well conserved among LRX proteins, the extensin domain is variable [17]. Many structural cell wall proteins, including extensins, are able to covalently crosslink in the cell wall and thereby influence mechanical properties [21–23]. For LRX1 of *Arabidopsis thaliana*, it was shown that the extensin domain crosslinks to cell wall components to anchor/insolubilize the protein and possibly properly position the LRR domain in the extracellular matrix [24, 25]. Expression of a truncated version of LRX1 lacking the extensin domain induces a dominant negative effect, resulting in an *lrx1*-like defect in root hair development. This suggests that the LRR domain binds and titrates out the interaction partner of the endogenous LRX1 and, hence, implies a binding partner of the LRR domain [24], the nature of which remains elusive.

The Arabidopsis genome codes for a family of eleven LRX-type proteins. *LRX1* and *LRX2* are paralogous genes and are predominantly expressed in root hairs where they function synergistically during cell development. *lrx1 lrx2* double mutants show a severe defect in root hair cell wall structures and growth, suggesting a role of LRX1 and LRX2 in cell wall formation [24, 26]. To better understand the function of LRX proteins during cell wall development, it is desirable to characterize the changes in cell wall structures and composition induced by mutations in *LRX* genes. Root hairs present a suboptimal cell type for these analyses due to their low abundance and atypical (for plant cells) tip growing mode of expansion. *LRX3*, *LRX4*, and *LRX5*, in contrast, are all expressed in roots and shoots, and cluster together in a phylogenetic tree based on amino acid homology of the encoded LRR domain. *LRX3* and *LRX4* are paralogs and share an almost identical expression profile [17]. Together, it can be hypothesized that these three LRX proteins have similar functions in overlapping tissues.

In this work, the characterization of *LRX3*, *LRX4*, and *LRX5* is described. Single, double, and triple mutants established using T-DNA insertion mutants reveal synergistic mutant phenotypes, suggesting a similar function of these three *LRX* genes. The changes in cell wall composition observed in the mutant lines compared to the wild type indicate that LRX proteins indeed have a function in cell wall formation. The lack of these proteins induces not only changes in cell wall structures but also strongly affects plant development implying that LRX proteins have an important function during cell (wall) development.

## Results

### LRX3, LRX4, and LRX5 are conserved LRR-extensin proteins

**Fig. 1:**
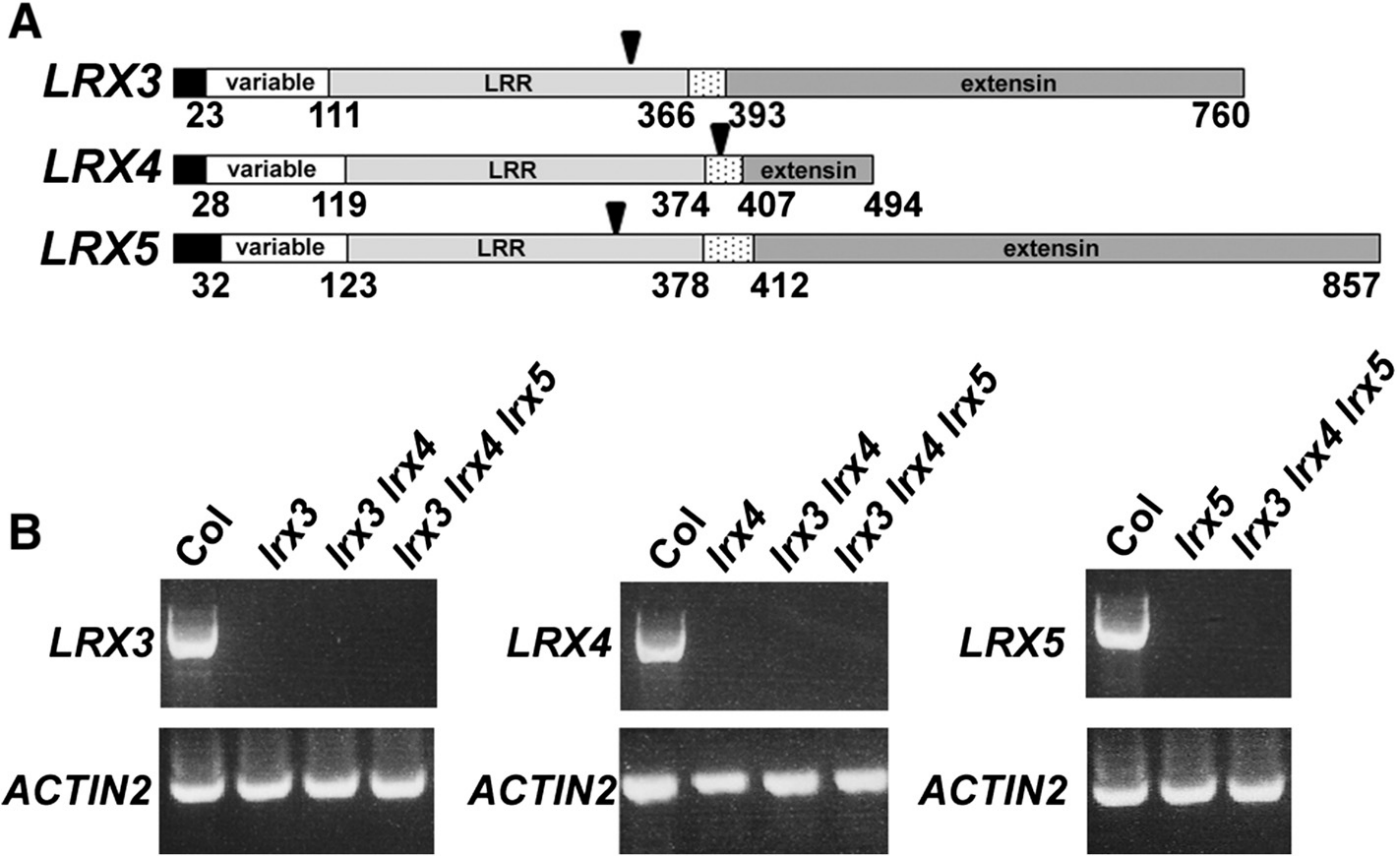
Protein structure of LRX3, LRX4, and LRX5 and gene expression. **a** Leucine-rich repeat extensin (LRX) proteins consist of a signal peptide for protein export (black), followed by a variable domain, nine complete leucine-rich repeats (LRR, grey), a Cys-rich hinge region (dotted), and a C-terminal extensin domain (dark grey) that show the typical Ser-Hyp_4_ motifs of hydroxyproline-rich glycoproteins. Numbers indicate amino acid positions, arrowheads the positions corresponding to the T-DNA insertions in the identified mutants. **b** RT-PCR on total RNA extracted from wild-type and mutant seedlings with gene-specific primers for each of the three *LRX* genes and *ACTIN2* as an internal control

LRX3, LRX4, and LRX5 proteins show the typical structure of leucine-rich repeat (LRR)-extensins (LRXs), including an N-terminal LRR domain and a C-terminal extensin (HRGP) domain. The N-terminal LRR domain is preceded by a domain that is variable amongst LRX proteins, while a Cys-rich hinge region separates the LRR and the extensin domains (Fig. 1a). An alignment of the LRR domains underlines the high degree of similarity between the three LRX proteins with 95% (LRX3 compared to LRX4), 86% (LRX3 compared to LRX5), and 85% (LRX4 compared to LRX5) (Additional file 1). As expected, the proteins encoded by the paralogous genes *LRX3* and *LRX4* [17] show the highest level of identity. By contrast, the extensin domain is much more variable amongst the three proteins, with a length of only 90 amino acids in LRX4 versus 367 and 445 amino acids in LRX3 and LRX5, respectively. The last 30 amino acid residues in all three proteins, however, show 75% identity (Additional file 1). The extensin domain contains the Ser-Hyp_4_ motif characteristic for this HRGP family. The tremendous length differences amongst the extensin domains are not necessarily indicative of functional differences since the extensin domains of LRX1 and LRX2 are functionally interchangeable despite limited homology [26].

### Mutations in *LRX* genes cause alterations in plant development

The importance of *LRX3*, *LRX4*, and *LRX5* was investigated by the identification and analysis of T-DNA insertion mutants. One mutant line was identified for each of the *LRX* genes (Salk_094400, GABI_017A08, and Salk_013968 for *LRX3*, *LRX4*, and *LRX5*, respectively) in which the coding region is interrupted (Fig. 1a). Since *LRX3* and *LRX4* are highly homologous paralogs [17] and thus likely to be functionally redundant, mutant analysis mainly focused on the *lrx3 lrx4* double mutant and the *lrx3 lrx4 lrx5* triple mutant. Homozygous mutants were obtained in all cases. To assess whether the mutant lines indeed fail to express the mutated genes, RT-PCR was performed on total RNA isolated from homozygous mutant seedlings. No RT-PCR product was obtained from any of the three mutant loci (Fig. 1b), confirming that the T-DNA insertions block gene expression and that the mutants most likely represent knock-out alleles.

**Fig. 2:**
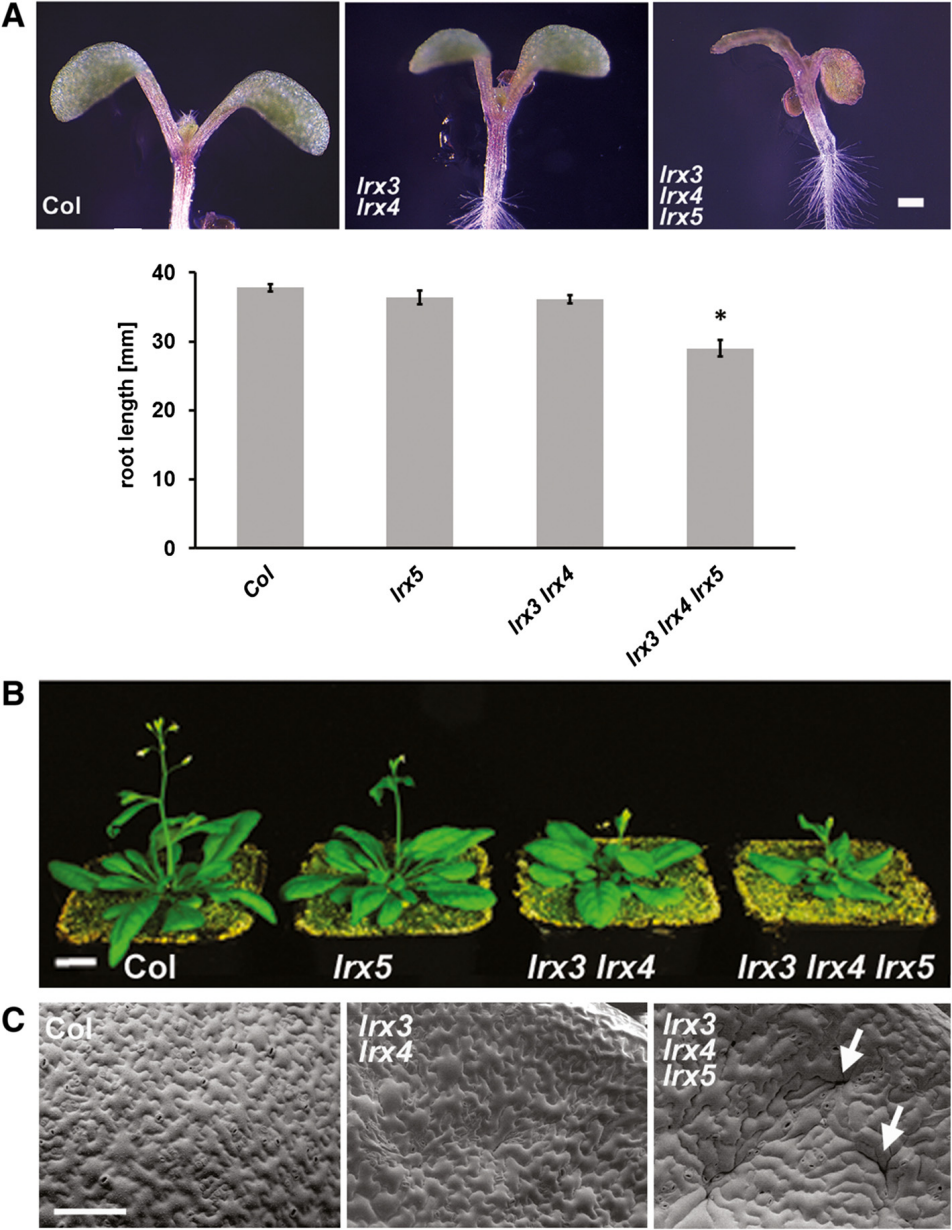
Mutations in *LRX* genes cause aberrant plant growth. **a** Cotyledons of 7 days-old seedlings are gradually smaller in the *lrx3 lrx4* double mutant and the *lrx3 lrx4 lrx5* triple mutant compared to the wild type (Col). Roots of the same seedlings are significantly shorter in the *lrx3 lrx4 lrx5* triple mutant. Error bars shown in the graph represent standard errors. Significance was tested by *T*-test; n ≥ 14, *: *P* < 0.05. **b** Mature plants of double and triple mutants reveal a reduction in growth compared to wild-type plants, whereas the *lrx5* single mutant grows comparable to the wild type. **c** Sinuous structures were observed in double and triple mutant cotyledons with occasional cracks (arrows) in the epidermis of the triple mutant. Bars: A = 0.5 mm; B = 10 mm; C = 100 μm

Since all three genes are expressed in seedlings and mature plants [17], both developmental stages were analyzed to identify potential mutant phenotypes. At the seedling stage, both double and triple mutant seedlings had smaller cotyledons. This phenotype was more pronounced in the triple mutant, where cotyledons were also less epinastic than in either the wild type or the *lrx3 lrx4* double mutant (Fig. 2a). Later during seedling development, the difference between the wild type, *lrx3 lrx4* double, and *lrx3 lrx4 lrx5* triple mutant was readily detectable in terms of size and shape, excluding that the mutants just grow slower than the wild type (Additional file 2A). Since the *lrx5* single mutant development was comparable to the wild type, the aggravated phenotype of the triple mutant suggests a synergistic activity of all three *LRX* genes during plant development. This synergistic effect was particularly detectable in root development where the *lrx3 lrx4* double mutant and the *lrx5* single mutant showed a root length similar to the wild type whereas the *lrx3 lrx4 lrx5* triple mutant roots were much shorter (Fig. 2a). The increasingly severe impairment of plant growth was observed also at older stages of development of the *lrx3 lrx4*, and *lrx3 lrx4 lrx5* mutants compared to the wild type, exemplified by the reduction in rosette leave size (Fig. 2b; Additional file 2B). Scanning electron microscopic analysis revealed a sinuous cotyledon surface in the double mutant that was not observed in the wild type. This effect was even more pronounced in the triple mutant lines, where crater-like structures developed with occasional cracks in the epidermal cell layer (Fig. 2c). The same phenotype in the epidermal surface was also observed in rosette leaves (Additional file 2C). It is important to note that the *lrx5* single mutant was comparable to the wild type at all stages of development and only in combination with the *lrx3 lrx4* mutations conferred an exaggerated growth defect indicative of a synergistic interaction between the *LRX* genes.

### Complementation of the *lrx* mutants

To confirm that the aberrant growth phenotypes observed in the *lrx3 lrx4* and *lrx3 lrx4 lrx5* mutants were induced by the T-DNA insertions, complementation experiments were performed. To this end, it was attempted to clone the wild-type copies of *LRX3*, *LRX4*, and *LRX5*. All three DNA constructs, however, were unstable in several different *E. coli* strains grown under different conditions. Most frequently, the extensin-coding region was truncated and could therefore not be propagated. Previous experiments had shown that extensin domains are interchangeable between different LRX proteins. The *lrx1* root hair mutant phenotype can be complemented with chimeric proteins containing the extensin domain of either LRX1 or LRX2 [26]. Therefore, the genomic clones of *LRX3* and *LRX4* containing the sequences of the promoter to the Cys-rich hinge domain-coding sequence (located in between the LRR- and extensin domains; Fig. 1a) were amplified and fused with the extensin-coding sequence and the terminator of *LRX1*. The resulting chimeric complementation constructs were referred to as *LRX3:LRR3-EXT1* and *LRX4:LRR4-EXT1*, respectively. For *LRX5*, this alternative cloning approach was also unsuccessful and thus, we abandoned complementation of the *lrx5* mutation.

**Fig. 3:**
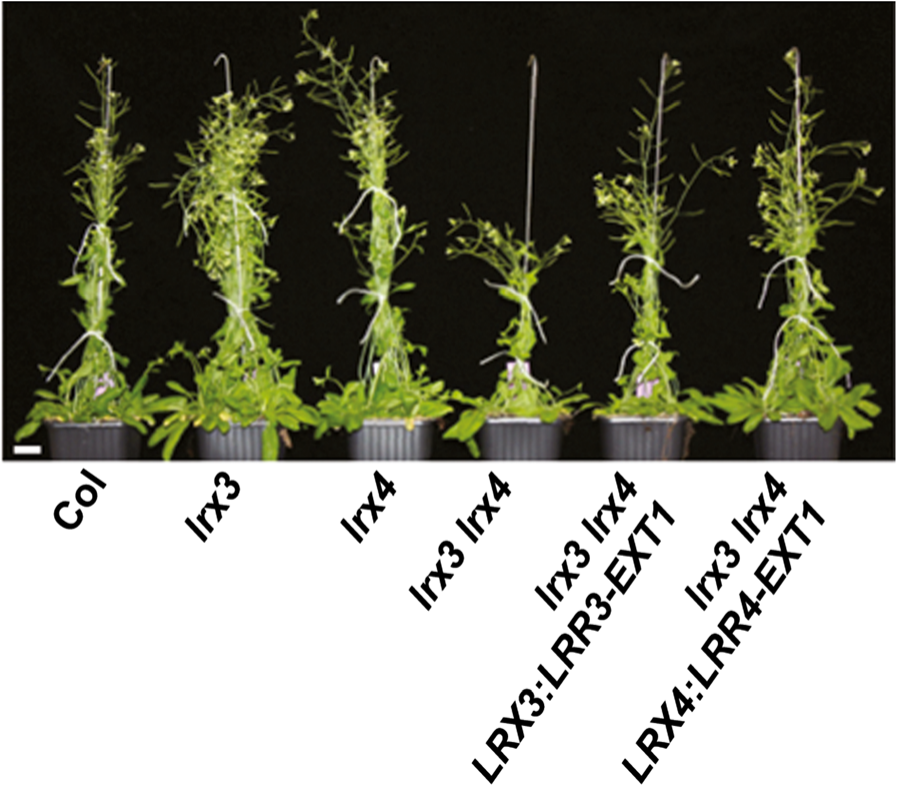
Mutations in *LRX* genes cause dwarfism. As exemplified by the *lrx3* and *lrx4* mutations, single mutants grow similar to the wild type whereas the *lrx3 lrx4* double mutant shows severely reduced growth that is alleviated by the *LRX3* and *LRX4* complementation constructs. Bar = 10 mm.

*LRX3:LRR3-EXT1* and *LRX4:LRR4-EXT1* were successfully transformed into the *lrx3 lrx4* double mutant. By contrast, transformation of the *lrx3 lrx4 lrx5* triple mutant resulted in poor seed set from which no transgenic seed could be recovered. Hence, complementation experiments were limited to the double mutant. Transgenic lines segregating in the T_2_ generation 3:1 for the selectable marker were analyzed using plant height as the parameter for complementation. While the *lrx3 lrx4* double mutant develops shorter inflorescences compared to the wild type or either of the single mutants, complementation with either *LRX3:LRR3-EXT1* or *LRX4:LRR4-EXT1* resulted in alleviation of the reduced plant height phenotype (Fig. 3), suggesting that the chimeric complementation constructs encode functional proteins and that the double mutant phenotype is indeed caused by the mutations in *LRX3* and *LRX4*.

### Changes in the ligno-cellulosic fraction, pectin, and xylan of the *lrx* mutants

**Fig. 4:**
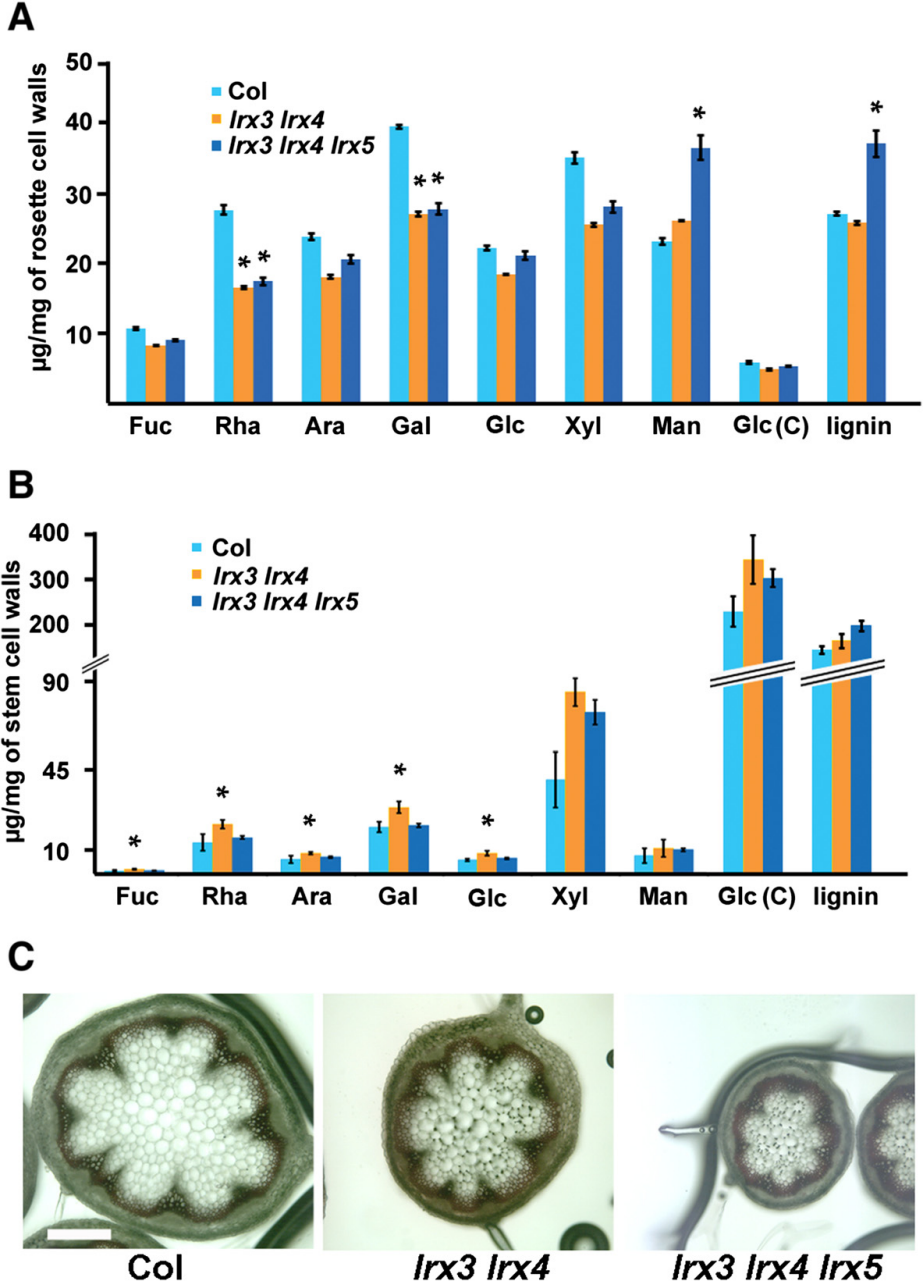
Cell wall composition analysis. Cell wall material was extracted from rosette leaves **a** and stem tissue **b** and monosaccharides were quantified. Error bars shown in the graph represent standard errors. Significance was tested by *T*-test; *n* = 3, *P < 0.05. **c** The degree of lignification was visualized in stem cross section using Wiesner staining. Bar = 500 μm.

Based on previous work, LRX proteins are assumed to have a role in cell wall development [24, 26]. Therefore, the composition and structures of wild-type and mutant cell walls were analyzed using various approaches. In a first step, the neutral sugar monosaccharide composition of cell wall material of the mutants and the wild type was performed. Analysis of rosette leaf alcohol insoluble residue (AIR) revealed a significantly (*T*-test, *P* < 0.05) reduced content in Rha and Gal in both mutants whereas Man increased in the triple mutant compared to the wild type. Similar to Man, lignification is increased in the triple mutant (Fig. 4a). In extracts of stem tissue, most sugars showed a significant (*T*-test, *P* < 0.05) increase in the double but not the triple mutant compared to the wild type (Fig. 4b). Even though the increase in lignin content in the mutants did not fulfill our criteria of significance, we still visualized lignification on stem sections. In agreement with the observed reduction in overall plant growth, cross-sections of the mutants were smaller in diameter than the wild type, which was also reflected by smaller cell sizes, and this effect was stronger in the triple mutant (Table 1). Compared to the wild type, the lignin-staining ring of (inter-)fascicular cells of the mutants covered a larger fraction of the entire stem section (Fig. 4c). To quantify this observation, the thickness of the interfascicular lignified ring was measured and compared to the full diameter. This confirmed that the lignified ring in relation to the stem diameter is larger in the mutants than in the wild type (Table 1).

**Table 1:** Quantification of stem diameter and degree of lignification in stem cross-sections

Genotype	Stem diameter [mm]	Lignin ring [mm]	% of ring to diameter
col	1.81	0.11	6
*lrx3 lrx4*	1.34 *	0.10	7.5 *
*lrx3 lrx4 lrx5*	1.01 *	0.09 *	9 *

Sections at the base were used for analysis. Significance was tested by *T*-test; n ≥ 15, *: significantly different from the wild type (col), *P* < 0.01

### CoMPP analysis of *lrx* mutants

To analyze the polysaccharide composition of the cell wall of the double and the triple mutant in more detail, a high-throughput characterization using Comprehensive Microarray Polymer Profiling (CoMPP; [27]) on rosette leaf and stem extracts was performed. Cell walls were sequentially extracted with diamino-cyclo-hexane-tetra-acetic acid (CDTA) and NaOH, to extract pectins and non-cellulosic polysaccharides, respectively, spotted on microarrays, and the intensity of the spot signals was quantified. CoMPP revealed significant changes in the relative intensities of JIM13 (recognizes arabinogalactan-proteins [AGPs]), JIM20 (recognizes extensins), LM11 (recognizes xylan), and LM13 (recognizes arabinan) (Table 2, Additional file 3). Samples extracted with CDTA and labeled with JIM13, JIM20, and LM13 revealed a reduction by up to one third in epitope detection levels. Leaf samples extracted with NaOH showed a reduced LM11 epitope intensity close to one half in the double mutant and a somewhat less in the triple mutant (Fig. 4a). LM13 (arabinan) epitopes were reduced up to 74% in *lrx3 lrx4* plants and up to 90% in *lrx3 lrx4 lrx5* mutants. The differential labelling observed between the wild type and the mutants does not systematically fit with the altered monosaccharide composition observed (Fig. 4)—for example in the case of some epitopes such as LM13 arabinan, this antibody will bind to only a subset of all arabinose-containing polymers in the cell walls. Together, these analyses confirm that the mutations lead to a modification of cell wall compositions in different organs.

**Table 2:** Glycome profiling of selected cell wall polysaccharide epitopes performed on cell wall extracts

Wash-ing	Mutant	Organ	JIM13 (AGP)	JIM20 (extensin)	LM11 (xylan)	LM13 (arabinan)
CDTA	wt	stem	90.04 ± 8.62	85.59 ± 8.41		97.44 ± 2.55
	*lrx3 lrx4*	stem	78.78 ± 0.48	75.97 ± 2.22		92.50 ± 2.05
	*lrx3 lrx4 lrx5*	stem	66.16 ± 13.46	58.93 ± 6.98		66.14 ± 4.35
NaOH	wt	leaf			95.54 ± 3.86	
	*lrx3 lrx4*	leaf			40.20 ± 2.80	
	*lrx3 lrx4 lrx5*	leaf			58.68 ± 16.16	
	wt	stem				49.91 ± 16.50
	*lrx3 lrx4*	stem				13.41 ± 7.81
	*lrx3 lrx4 lrx5*	stem				5.22 ± 5.17

Only significant differences between wild type and mutants are shown. Values are mean signal intensities ± standard deviation of three independent extracts. Detailed data is shown in the Additional file 3

## Discussion

Leucine-rich repeat extensins (LRX) are extracellular proteins involved in cell wall assembly. This assumption was based on the previous characterization of mutants of the Arabidopsis *LRX1* and *LRX2* that revealed a defect in the cell wall ultrastructure [24, 26]. The analysis of *LRX3*, *LRX4*, and *LRX5* support this proposed function since mutations in these genes lead to aberrant cell wall composition. The alterations ultimately impact cell growth properties, resulting in generally reduced plant growth. The repetitive nature of the extensin-coding region of *LRX* genes made the cloning strategy difficult, in particular of *LRX5*, which we were unable to clone and utilize for complementation experiments. For *LRX3* and *LRX4*, chimeric constructs containing the *LRX1* extensin-coding region had to be used. This resulted in functional proteins, consistent with the previous domain swap experiments, which showed that the extensin domains of LRX1 and LRX2 are interchangeable [26]. The complementation of the *lrx3 lrx4* double mutant with *LRX3* and *LRX4* combined with the analysis of the double/triple mutants provide good evidence that the mutant phenotypes observed are indeed caused by the mutations in these three *LRX* genes. Even though complementation of the *lrx5* mutant was not possible due to technical difficulties, the synergistic effect of *lrx5* and *lrx3 lrx4* in the context of the *lrx3 lrx4 lrx5* triple mutant implies comparable activities of the affected proteins.

Several different cell wall structures are modified in the *lrx3 lrx4* double and *lrx3 lrx4 lrx5* triple mutants. Also, these changes differ between rosette leaves and stem tissue. Rosette leaves show reduced levels of the monosaccharides Rha and Gal. In stem tissue, an increase in most neutral sugars was found for the *lrx3 lrx4* double mutant. Since Rha and Gal are found in pectin, tissues with a high proportion of pectin-rich primary cell walls [28], such as rosette leaves, are more likely to reveal a difference in pectin content. Interestingly, LM13, which binds longer oligoarabinosides thought to be part of the pectin fraction, gives a strongly reduced signal in stems but not rosette leaf extracts. Since this is not reflected in a reduced Ara content, Ara-containing structures not detected by LM13 are probably also modified. In stems, the LM13 epitope is mostly abundant in epidermal walls and implicated in responses to mechanical stress [29, 30]. It is likely that the changes in cell wall structures affect the mechanical properties in the stem, which in turn also modifies the accumulation of LM13-detected pectic arabinan. In rosette leaf cell walls of the mutants, mannose is the only neutral sugar that shows an increase in abundance in *lrx* mutants. Mannose forms several types of mannan polymers which represent hemicelluloses that are particularly abundant in secondary cell walls and woody tissue [31, 32]. In the *lrx3 lrx4 lrx5* triple mutant, however, a change in mannan and lignin is not found in stem but only in rosette leaves. Whether the increase of mannose and lignin in rosette leaves is based on a common regulation remains to be shown.

Depending on the cellular function, tissues respond differently to changes in growth properties. This would provide an explanation for the observed divergence in structural adaptation of cell walls of leaf and stem tissues to mutations in *lrx3*, *lrx4*, and *lrx5*. Alternatively, changes in the composition of the cell wall can trigger a number of additional, secondary modifications as an attempt of the plant to directly compensate for alterations in the extracellular matrix [11]. Compensatory changes might depend on the severity of defects in cell wall formation, which would explain the observation of certain changes in stem tissue only in the *lrx3 lrx4* double mutant but not the *lrx3 lrx4 lrx5* triple mutant. Reducing cellulose content by the *procuste1* or *fei1 fei2* mutants causes an increase in lignin content [16, 33]. Other changes than a reduction in cellulose content may also induce increased lignification, as the level of cellulose is not affected by the *lrx* mutations, yet lignification is elevated in rosette leaves. In stems, the fraction of the lignin ring relative to the stem diameter is increased. This, however, is not reflected by an altered lignin content, suggesting that the distribution of lignin is modified. Xylan is associated with cellulose and mainly found in secondary cell walls. In CoMPP experiments, the binding of the anti-xylan antibody LM11 is decreased in leaf tissue. The LM11 antibody recognizes β1-4 linked Xyl and also arabinoxylan [34]. The xylan backbone is frequently substituted with side chains of either glucuronic acid (GlcA) or methyl-GlcA, and a reduction in these substitutions changes xylan structure and can cause a dwarf growth phenotype [35]. Modified antibody labelling might be induced by either changes in the overall xylan structure or epitope accessibility. Future experiments with a variety of anti-xylan antibodies might provide better information on the potential changes in the xylan structure induced by the *lrx* mutations.

The anti-extensin antibody JIM20 also shows a reduction in labelling in the mutants compared to the wild-type. The Arabidopsis genome contains over 20 genes that encode extensin proteins [36], a number of which are expressed in stem tissue. The contribution of each of these proteins to JIM20 labelling is not known. Hence, it is possible that the absence of the three LRX proteins causes a reduction in labelling since they all represent potential epitopes of JIM20. Extensins belong to the class of HRGPs and are known to influence physical properties of cell walls by their ability to form an insolubilized, covalent network in cell walls [37–39]. Root hair formation appears to be particularly sensitive to changes in this protein network since interfering with proline hydroxylation of extensins causes defects in root hair development [40]. RSH/EXT3 has been shown to be important for cell wall formation during cytokinesis. With its propensity to self-aggregate, RSH/EXT3 has been proposed to form a scaffold for the deposition of new cell wall material [36, 41]. In the context of LRX proteins, however, the extensin domain appears to have an anchoring function. The insolubilization of LRX1 is not a default reaction but developmentally regulated. Also, overexpression of the extensin domain of LRX1 does not alleviate the *lrx1* root hair formation defect, suggesting that LRX proteins are not primarily reinforcing the cell wall but the extensin domain might rather position the protein within the cell wall [24, 25].

The numerous changes in cell wall structures found in the *lrx* mutants reflects a general problem in the analysis of cell wall mutants: the difficulty to distinguish between primary effects of the mutations under investigation and the secondary effects induced by the plant with the aim of compensating the primary changes. In the *cesa6* mutant *procuste1*, at least some of the compensatory changes in cell wall structures are induced by the receptor-kinase THESEUS1, which plays a role in cell wall integrity sensing. The *the1* mutation blocks compensatory modifications, resulting in a partial suppression of the *procuste1* mutant phenotype [10]. It will be interesting to see whether some of the cell wall modifications observed in the *lrx* mutants are also induced by THESEUS1 and thus whether the introduction of the *the1* mutation in the *lrx* mutant background might help identifying the primary changes induces by the mutations in *LRX* genes.

The changes in cell wall structures observed in the *lrx3 lrx4* and *lrx3 lrx4 lrx5* mutants do not lead to a conclusive picture on the function of LRX proteins during cell wall formation. The interaction partner of LRX proteins would be very informative in this context but remains currently unknown. LRX proteins do not appear to serve as transmembrane receptor proteins since they lack a transmembrane domain. On the other hand, they are anchored in the cell wall via their extensin domain [24, 25]. The identification of the interacting partner(s) should provide a better picture on the orientation of the LRR domain of LRX proteins. Hypothetically, the LRR domain might interact with a membrane-bound component and thus serve in establishing a membrane-cell wall signaling continuum with the goal of controlling the deposition of new cell wall material. This would provide an alternative explanation for the pleiotropic changes in cell wall structure and composition observed in the *lrx* mutants. Alternatively, the LRR domain of LRX proteins might influence extracellular enzymatic activity. One class of LRR-containing proteins known to bind and inhibit cell wall degrading enzymes are endopolygalacturonase-inhibiting proteins (PGIPs) which are involved in plant development as well as pathogen defense [42, 43]. The LRR domain of these proteins contains eleven repeats, which is very similar to the ten and a half repeats of LRX proteins [17]. Whether this reflects a functional similarity is not yet clear, particularly since the LRR domains of LRXs and PGIPs share limited sequence homology. Hence, LRX proteins might be involved in either the regulation of enzymatic activities in the cell wall or the recruitment of enzymes to the appropriate location in the cell wall. It can be speculated that the function of LRX proteins is related to pectins, since mutations modifying pectin structures were found to suppress the *lrx1* root hair phenotype [44, 45].

## Conclusions

LRX3, LRX4, and LRX5 are homologous LRR-extensin proteins found in overlapping tissues and have similar functions. Mutations in these *lrx* genes induce growth phenotypes implying that LRX proteins are indispensable for proper plant development. Since LRX proteins localize to the cell wall, the identification of numerous changes in cell wall structures of *lrx3*, *lrx4*, and *lrx5* mutant lines support the assumption that LRX proteins are involved in cell wall development. Additional analyses are necessary to unravel the exact function of LRX proteins in this process.

## Methods

### Plant material and growth

For experiments wild-type *Arabidopsis thaliana* (ecotype Col-0) and the mutants *lrx3* (At4g13340; Salk_094400), *lrx4* (At3g24480; GABI_017A08), and *lrx5* (At4g18670; Salk_013968) were used. T-DNA insertions are located 752 bp, 1173 bp and 898 bp downstream of the start codon in *lrx3*, *lrx4*, and *lrx5*, respectively.

Seeds were obtained from the NASC European Arabidopsis stock center. Seeds were surface sterilized (1% sodium hypochlorite, 0.03% TritonX-100) and stratified 2–4 days at 4 °C. Seeds were plated on 1/2 Murashige and Skoog medium (0.6% phytagel (Sigma), 2% sucrose) and grown in growth chambers with 16 h light, 8 h dark cycles at 22 °C.

### DNA primers, constructs and plant transformation

For the complementation constructs *LRX3:LRR3 EXT1* and *LRX4:LRR4 EXT1*, the promoter and coding sequence encoding the hinge region of *LRX3* and *LRX4* were amplified by PCR with the following primers to introduce a PstI site at the 3′ end coding for the hinge region: LRX3F: 5′-TCATATGTGCTGTAGATGATTGGG-3′; LRX3R: 5′-CTGCAGTTTACCGGCGGACGAGACAAAAACG-3′; LRX4F: 5′-ACCCTCTAGCCTTTATATATTTATAG-3′; LRX4R: 5′-CTGCAGTCCACCGAAGGCCGTGACAAGAAAG-3′. The PCR fragments were cloned into the pSC PCR cloning vector (Stratagene) and correct clones were cut out by ApaI/PstI and cloned into a vector containing the *LRX1* extensin-coding region and terminator [26] opened with the same enzymes. For plant transformation the constructs were cloned into pBART27 [46, 47] with NotI and plants were transformed and T_1_ transformants selected as described [48]. T_2_ seeds obtained from the primary transformants were sterilized and sown on BASTA plates and resistant seedlings were transferred to soil for propagation.

### Pavement cell analysis and SEM analysis

Imprinting of pavement cells was done following Horiguchi et al. [49] and observed with a Zeiss AX10 microscope. Scanning Electron Microscope (SEM) analysis of cotyledons and rosette leaves was done as described by Baumberger et al. [26].

### Comprehensive microarray polymer profiling (CoMPP) and monosaccharide analysis

Preparation of AIR (alcohol insoluble residue) was done as follows: Plant materials (stem and rosette leaves of adult plants) were ground in liquid nitrogen, washed with 80% EtOH three times and subsequently washed in 100% acetone, and CoMPP was performed as described in Moller et al. [27]. Quantification was done with biological triplicates and the average values and standard deviations are indicated in the tables.

### Monosaccharide composition and linkage analysis of polysaccharides

The analyses of polysaccharides were performed on an AIR prepared as follows. One hundred mg (FW) of ground rosette leaf or stem tissue of adult plants were washed twice in 4 volumes of absolute ethanol for 15 min, then rinsed twice in 4 volumes of acetone at room temperature for 10 min and left to dry under a fume hood overnight at room temperature. Neutral monosaccharide composition analysis was performed on 5 mg of dried AIR after hydrolysis in 2.5 M TFA for 1.5 h at 100 °C as described in [50]. To determine the cellulose content, the residual pellet obtained after the monosaccharide analysis was rinsed twice with ten volumes of water and hydrolysed with H_2_SO_4_ as described [51]. The released glucose was diluted 500 times and then quantified using an HPAEC-PAD chromatography as described in [50]. Quantification was done with biological triplicates and the average values and standard errors are indicated in the graphs.

### Lignin quantification and visualization

One hundred mg (FW) of dry ground leaf were washed twice in 3 mL of water at 80 °C for 15 min, twice in 3 mL of ethanol at 80 °C for 15 min and once in 3 mL acetone at room temperature for 10 min and left to dry under a fume hood overnight at room temperature. The following protocol is adapted from Fukushima and Hatfield [52]. Lignin from the prepared cell wall residue was solubilized in 1 mL of acetyl bromide solution (acetyl bromide/acetic acid (1/3, V/V)) in a glass vial at 55 °C for 2.5 h under shaking. Samples was then let to cool down at room temperature and 1.2 mL of NaOH 2 M/Acetic acid (9/50 V/V) was added in the vial. One hundred μL of this sample was transferred in 300 μL of 0.5 M hydroxylamine chlorhydrate and mixed with 1.4 mL of acetic acid. The A_280_ absorbance of the samples was measured. Lignin content was calculated using the following formula: %lignin = 100 x (A280 x V reaction x V dilution) / (20 x V sample solution x m sample in mg). In stem cross-sections lignin was visualized by Wiesner staining. Quantification was done with biological triplicates and the average values and standard errors are indicated in the graphs.

### Accession numbers

The accession numbers of the genes analyzed in this study are as follows: *LRX3*: At4g13340; *LRX4*: At3g24480; *LRX5*: At4g18670.

## Additional files

Additional file 1:
**Alignment of LRX3, LRX4 and LRX5 proteins.** Alignment of the full-length proteins by ClustalW revealed high homology in the N-terminal variable domain as well as the leucine-rich repeat domain (flanked by arrowheads). The extensin domains are very different between the proteins, except the last 35 amino acids which are again well conserved. The Pro residues encoded in the extensin domain are posttranslationally modified to Hyp. Identical, conserved, and similar positions in the alignment are indicated by asterisks, colons, or single dots, respectively. Additional file 2:
**Growth defect phenotypes of the** ***lrx*** **mutants.** (A) While the wild type and *lrx5* single mutant are comparable in size, the double and triple mutant seedlings grow gradually smaller, indicating a synergistic interaction between the *lrx* mutations. (B) Rosette leaves of the mutants also show a gradually reduced leave area compared to the wild type. Error bars shown in the graphs represent standard errors. Significance was tested by T-test; n=6, *: P<0.05. (C) Compared to the even surface of wild-type (Col) rosette leaves, double and triple mutants frequently developed uneven, sinuous surfaces with occasional cracks (arrows) in the epidermis of the triple mutant. Bars: A= 5 mm; B= 10 mm; C= 300 μm. Additional file 3:
**Glycome profiling of selected cell wall polysaccharide epitopes.** Biological triplicates of cell wall extracts of the wild type, the *lrx3 lrx4*, and the *lrx3 lrx4 lrx5* mutants were washed with either CDTA or NaOH and epitope abundance was measured by quantification of antibody binding and expressed here as relative spot intensity (corrected to 100). The monoclonal antibodies used and bound epitopes are listed. The upper half of the table represents the mean values of technical triplicates of each extract, the lower half lists the standard deviation in the technical triplicates.

## Abbreviations

HRGP: Hydroxyproline-rich glycoprotein
LRX: Leucine-rich repeat extensin
LRR: Leucine-rich repeat
PGIP: Polygalacturonase inhibitor
AGP: Arabinogalactan protein
AIR: Alcohol insoluble residue
CoMPP: Comprehensive microarray polymer profiling
CDTA: Diamino-cyclo-hexane-tetra-acetic acid
