# Supplementary material for: Arabidopsis leucine-rich repeat extensin (LRX) proteins modify cell wall composition and influence plant growth
Source: BMC Plant Biol. 2015 Jun 24;15:155. doi: 10.1186/s12870-015-0548-8 (PMC4477543; doi:10.1186/s12870-015-0548-8)
Supplement: Additional file 1: — Alignment of LRX3, LRX4 and LRX5 proteins. Alignment of the full-length proteins by ClustalW revealed high homology in the N-terminal variable domain as well as the leucine-rich repeat domain (flanked by arrowheads). The extensin domains are very different between the proteins, except the last 35 amino acids which are again well conserved. The Pro residues encoded in the extensin domain are posttranslationally modified to Hyp. Identical, conserved, and similar positions in the alignment are indicated by asterisks, colons, or single dots, respectively. [file 12870_2015_548_MOESM1_ESM.pdf]

Additional File 1

```
LRX3          -----MK----KTIQILLFFFFLINLTNALSISSDGGVLSDNEVRHIQRRQLLE----FA
LRX4          MKNNTTQ----SLLLLLLFFFFFFEISHSLSISSN-APLSDTEVRFIQRRQLLYYRDEFG
LRX5          MKTKMMMKNTSLIFVLLFITFFFTSISYSLSLTF-NGDLSDNEVRLITQRQLLYFRDEFG
                           : :*:: **: .:: :**::   . ***.*** * :****     *.
LRX3          ERSVKITVDPSLNFENPRLRNAYIALQAWKQAILSDPNNFTSNWIGSNVCNYTGVFCSPA
LRX4          DRGENVTVDPSLIFENPRLRSAYIALQAWKQAILSDPNNITVNWIGSNVCNYTGVFCSKA
LRX5          DRGENVDVDPSLVFENPRLRNAYIALQAWKQAILSDPNNFTTNWIGSDVCSYTGVYCAPA
              :*. :: ***** *******.******************:* *****:**.****:*: *

LRX3          LDNRKIRTVAGIDLNHADIAGYLPEELGLLSDLALFHVNSNRFCGTVPHRFNRLKLLFEL
LRX4          LDNRKIRTVAGIDLNHADIAGYLPEELGLLTDLALFHVNSNRFCGTVPHKFKQLKLLFEL
LRX5          LDNRRIRTVAGIDLNHADIAGYLPQELGLLTDLALFHINSNRFCGTVPHRFNRLKLLFEL
              ****:*******************:*****:******:***********:*::*******

LRX3          DLSNNRFAGKFPTVVLQLPSLKFLDLRFNEFEGTVPKELFSKDLDAIFINHNRFRFELPE
LRX4          DLSNNRFAGKFPTVVLHLPSLKFLDLRFNEFEGTVPKELFSKNLDAIFINHNRFRFELPE
LRX5          DLSNNRFAGIFPTVVLQLPSLKFLDLRFNEFEGPVPRELFSKDLDAIFINHNRFRFELPD
              ********* ******:**************** **:*****:****************:

LRX3          NFGDSPVSVIVLANNRFHGCVPSSLVEMKNLNEIIFMNNGLNSCLPSDIGRLKNVTVFDV
LRX4          NFGDSPVSVIVLANNHFHGCIPTSLVEMKNLNEIIFMNNGLNSCLPADIGRLKNVTVFDV
LRX5          NLGDSPVSVIVVANNHFHGCIPTSLGDMRNLEEIIFMENGFNSCLPSQIGRLKNVTVFDF
             *:*********:***:****:*:**  :*:**:*****:**:*****::***********.

LRX3          SFNELVGPLPESVGEMVSVEQLNVAHNMLSGKIPASICQLPKLENFTYSYNFFTGEAPVC
LRX4          SFNELVGPLPESVGGMVEVEQLNVAHNLLSGKIPASICQLPKLENFTYSYNFFTGEAPVC
LRX5          SFNELVGSLPASIGGMVSMEQLNVAHNRFSGKIPATICQLPRLENFTFSYNFFTGEPPVC
              ******* ** *:* **.:******** :******:*****:*****:******** ***

LRX3          LRLPEFDDRRNCLPGRPAQRSPGQCKAFLSRPPVNCGSFSCGRSVSPRPPVVTPLPPPSL
LRX4          LRLSEFDDRRNCLPGRPAQRSSRQCSAFLSRPSVDCGSFGCGRSVVKPSPPIVALPPPPP
LRX5          LGLPGFDDRRNCLPARPAQRSPGQCAAFSSLPPVDCGSFGCGRSTRPPV--VVPSPPTTP
              * *  *********.******  ** ** * * *:****.****.      :.  **

LRX3          -PSPPPPAPIFSTPPTLTSPPPPSPPPP-------V------------------------
LRX4          -PSPPLPPPVY-------------------------------------------------
LRX5          SPGGSPPSPSISPSPPITVPSPPTTPSPGGSPPSPSIVPSPPSTTPSPGSPPTSPTTPTP
              *.   * *

LRX3          --------------YSPPP-------------PP-------PPPPPVYSPPP--------
LRX4          ---------------SPPP-------------S-----------PPVFSPP---------
LRX5          GGSPPSSPTTPTPGGSPPSSPTTPTPGGSPPSSPTTPSPGGSPPSPSISPSPPITVPSPP
                             ***                           *  **

LRX3          ------------------------------------------------------------
LRX4          ------------------------------------------------------------
LRX5          STPTSPGSPPSPSSPTPSSPIPSPPTPSTPPTPISPGQNSPPIIPSPPFTGPSPPSSPSP


LRX3          -PPPPPPPPPVYSPPPPPPPPPPPPPVYSPPPPSPPPPPPPVYSP-PPPPPPPPPPPVYS
LRX4          ------PSPPVYSPPPPPS------IHYS---------------S-P-------------
LRX5          PLPPVIPSPPIVGPTPSSPPPSTPTPVYSPPPPSTGYPPPPPFTGYSPPSPPPPPPPTFS
                    * **: .* *           **

LRX3          ------PPPPPVYS---------------SPPPPPSPAPTPVYCTRPPPPPPHSPPPPQF
LRX4          -------PPP--------------------------------------------------
LRX5          PSPSIPPPPPQTYSPFPPPPPPPPQTYYPPQPSPSQPPQSPIYGTPPPSPIPYLPSPPQF
                     ***

LRX3          S-PPPPEPYYYSSPPPPHSSPPPHSPPPPHSPPPPIYPYLSPPPPPTPVSSPPPTPVYSP
LRX4          ------------------------------------------------------------
LRX5          ASPPPPAPYYYSSPQPPPP---PH---YSLPPPTPTYHYISPPPPPTPIHSPPPQ-----


LRX3          PPPPPCIEPPPPPPPCIEYSPPPPPPVVHYSSPPPPPVYYSSPPPPPVYY-SSPPPPPPVH
LRX4          ------------------------------------------------------------
LRX5          ----------SHPPCIEYSPPPPPTVHYNPPPPPSPAHYSPPPSPPVYYYNSPPPPPAVH
```

```
LRX3      YSSPPPPEVHYHSPPPSPVHYSSPPPPPSAPCEESPPPAPVVHHSPPPPMVHHSPPPPVI
LRX4      ---------------------------------------------------------PVH
LRX5      YSP-----------------------------------------------------PPPPVI
                                                                   **

LRX3      HQSPPPPSPEYEGPLPPVIGVSYASPPPPPPFY
LRX4      HSSPPPPSPEFEGPLPPVIGVSYASPPPPPPFY
LRX5      HHSQPPPPPIYEGPLPPIPGISYASPPPPPPFY
          * * *** * :******: *:************
```

Supplementary Fig. S1 Alignment of LRX3, LRX4 and LRX5 proteins.

Alignment of the full-length proteins revealed high homology in the N-terminal variable domain as well as the Leucine-rich repeat domain (flanked by arrowheads). The extensin domains very different between the proteins, except the last 35 amino acids which are again well conserved.
