# Supplementary figures and images for: Arabidopsis leucine-rich repeat extensin (LRX) proteins modify cell wall composition and influence plant growth
Source: BMC Plant Biol. 2015 Jun 24;15:155. doi: 10.1186/s12870-015-0548-8 (PMC4477543; doi:10.1186/s12870-015-0548-8)

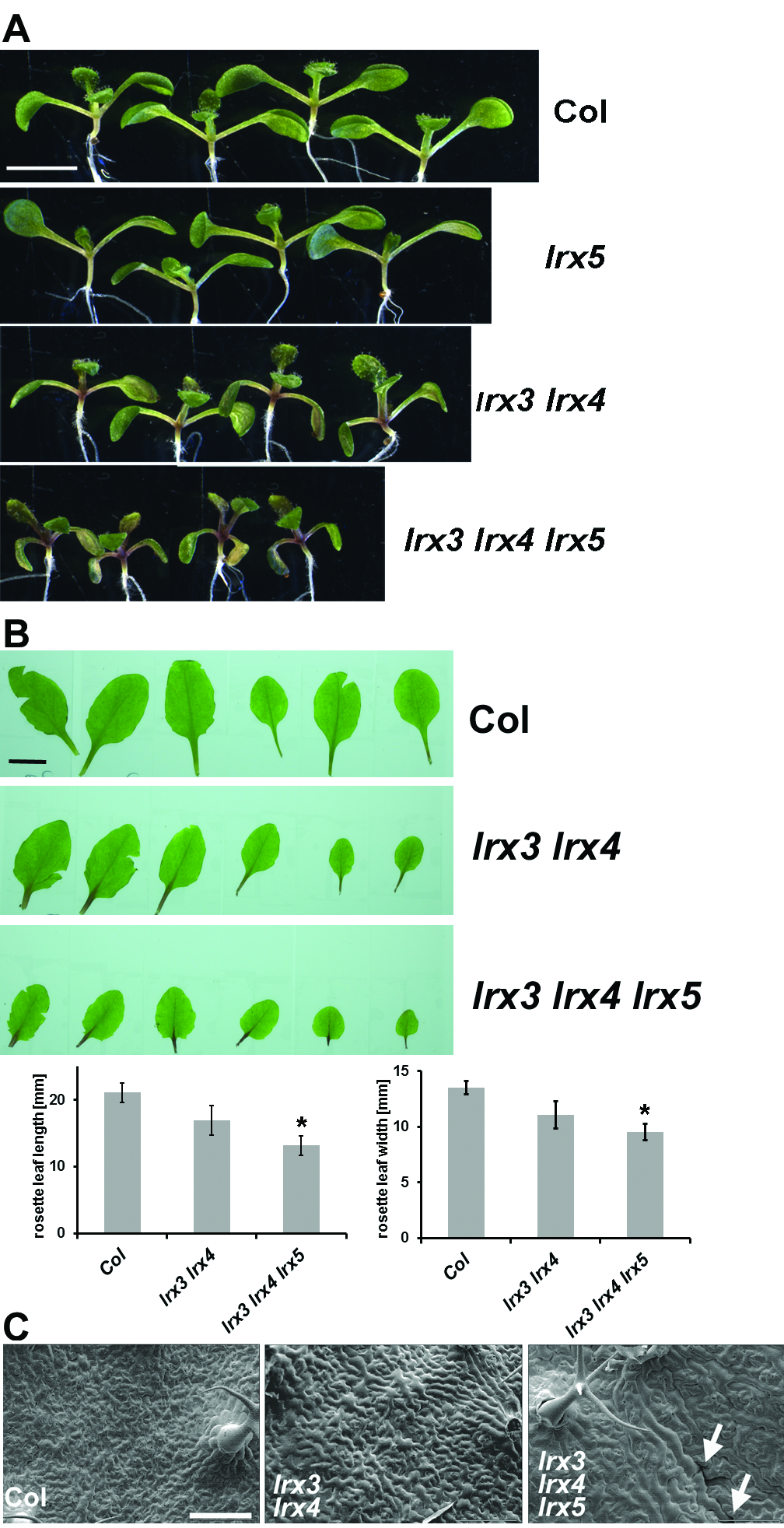

Supplement: Additional file 2: — Growth defect phenotypes of the lrx mutants. (A) While the wild type and lrx5 single mutant are comparable in size, the double and triple mutant seedlings grow gradually smaller, indicating a synergistic interaction between the lrx mutations. (B) Rosette leaves of the mutants also show a gradually reduced leave area compared to the wild type. Error bars shown in the graphs represent standard errors. Significance was tested by T-test; n=6, *: P<0.05. (C) Compared to the even surface of wild-type (Col) rosette leaves, double and triple mutants frequently developed uneven, sinuous surfaces with occasional cracks (arrows) in the epidermis of the triple mutant. Bars: A= 5 mm; B= 10 mm; C= 300 μm. [file 12870_2015_548_MOESM2_ESM.tiff]
