# Supplementary material for: Arabidopsis leucine-rich repeat extensin (LRX) proteins modify cell wall composition and influence plant growth
Source: BMC Plant Biol. 2015 Jun 24;15:155. doi: 10.1186/s12870-015-0548-8 (PMC4477543; doi:10.1186/s12870-015-0548-8)
Supplement: Additional file 3: — Glycome profiling of selected cell wall polysaccharide epitopes. Biological triplicates of cell wall extracts of the wild type, the lrx3 lrx4, and the lrx3 lrx4 lrx5 mutants were washed with either CDTA or NaOH and epitope abundance was measured by quantification of antibody binding and expressed here as relative spot intensity (corrected to 100). The monoclonal antibodies used and bound epitopes are listed. The upper half of the table represents the mean values of technical triplicates of each extract, the lower half lists the standard deviation in the technical triplicates. [file 12870_2015_548_MOESM3_ESM.pdf]

Additional File 3

average

washing	mutant	organ	HG LM19	AGP JIM13	extensin JIM20	galactan LM5	arabinan LM6	xylan LM11	arabinan LM13	xyloglucan LM15
CDTA	wt	leaves	87.60	0.00	54.85	59.81	7.06	0.00	18.41	6.06
	3,4	leaves	69.18	0.00	54.79	35.91	0.00	0.00	18.15	0.00
	3,4,5	leaves	72.99	3.97	64.18	43.90	2.33	0.00	31.35	2.99
	wt	stem	74.66	90.04	85.59	65.23	1.83	14.50	97.44	2.74
	3,4	stem	72.91	78.78	75.97	83.27	7.05	23.59	92.50	8.99
	3,4,5	stem	76.28	66.16	58.93	93.33	4.80	21.22	66.14	9.99
NaOH	wt	leaves	0.00	19.80	77.08	58.59	83.74	95.54	7.72	73.39
	3,4	leaves	7.74	22.29	71.68	64.05	76.04	40.20	7.47	63.60
	3,4,5	leaves	6.35	20.00	83.02	72.45	74.06	58.68	9.75	69.33
	wt	stem	0.00	40.86	30.43	58.03	87.15	52.43	49.91	82.47
	3,4	stem	0.00	53.30	36.55	43.83	68.14	37.98	13.41	60.50
	3,4,5	stem	0.00	35.84	34.28	47.98	72.72	45.01	5.22	62.76

standard dev.

washing	mutant	organ	HG LM19	AGP JIM13	extensin JIM20	galactan LM5	arabinan LM6	xylan LM11	arabinan LM13	xyloglucan LM15
CDTA	wt	leaves	13.96	0.00	7.71	2.29	6.89	0.00	2.86	5.66
	3,4	leaves	15.01	0.00	30.58	25.45	0.00	0.00	11.08	0.00
	3,4,5	leaves	3.62	6.88	9.98	10.08	4.04	0.00	6.38	5.19
	wt	stem	5.47	8.62	8.41	5.56	3.17	1.58	2.55	4.74
	3,4	stem	7.11	0.48	2.22	2.96	1.76	1.99	2.51	4.36
	3,4,5	stem	13.37	13.46	6.98	7.30	4.66	6.44	4.35	4.22
NaOH	wt	leaves	0.00	6.43	8.06	7.42	14.15	3.86	1.56	10.97
	3,4	leaves	2.18	13.14	17.67	5.37	0.58	2.80	1.91	6.37
	3,4,5	leaves	1.38	7.29	17.34	10.51	3.78	16.16	5.03	14.13
	wt	stem	0.00	3.93	9.59	0.77	7.46	3.09	16.50	15.46
	3,4	stem	0.00	4.81	13.51	4.41	7.24	6.49	7.81	3.38
	3,4,5	stem	0.00	0.86	16.03	19.19	11.41	6.70	5.17	10.48

Additional File 2 Glycome profiling of selected cell wall polysaccharide epitopes.

Biological triplicates of cell wall extracts of the wild type, the *lrx3 lrx4*, and the *lrx3 lrx4 lrx5* mutants were washed with either CDTA or NaOH and epitope abundance was measured by quantification of antibody binding and expressed here as relative spot intensity (corrected to 100). The monoclonal antibodies used and bound epitope are listed. The upper half of the table represents the mean values of technical triplicates of each extract, the lower half lists the standard deviation in the technical triplicates.
